# Comparing unplanned and potentially planned home deaths: a population-based cross-sectional study

**DOI:** 10.1186/s12904-018-0323-3

**Published:** 2018-05-02

**Authors:** Camilla Kjellstadli, Bettina Sandgathe Husebø, Hogne Sandvik, Elisabeth Flo, Steinar Hunskaar

**Affiliations:** 1 Research Group for General Practice, Department of Global Public Health and Primary Care, University of Bergen, PO box 7804, N-5018 Bergen, Norway; 2 Centre for Elderly and Nursing Home Medicine, Department of Global Public Health and Primary Care, University of Bergen, PO box 7804, N-5018 Bergen, Norway; 3 Bergen Municipality, Bergen, Norway; 4 National Centre for Emergency Primary Health Care, Uni Research Health, Kalfarveien 31, N-5018 Bergen, Norway; 5 Department of Clinical Psychology, University of Bergen, PO box 7804, N-5018 Bergen, Norway

**Keywords:** Home death, Place of death, Death/epidemiology, Death/statistics and numerical data, Death/classification, Cross-sectional studies, Cause of death, Terminal care, Registries, Retrospective studies

## Abstract

**Background:**

There is little research on number of planned home deaths. We need information about factors associated with home deaths, but also differences between planned and unplanned home deaths to improve end-of-life-care at home and make home deaths a feasible alternative. Our aim was to investigate factors associated with home deaths, estimate number of potentially planned home deaths, and differences in individual characteristics between people with and without a potentially planned home death.

**Methods:**

A cross-sectional study of all decedents in Norway in 2012 and 2013, using data from the Norwegian Cause of Death Registry and National registry for statistics on municipal health and care services. We defined planned home death by an indirect algorithm-based method using domiciliary care and diagnosis. We used logistic regressions models to evaluate factors associated with home death compared with nursing home and hospital; and to compare unplanned home deaths and potentially planned home deaths.

**Results:**

Among 80,908 deaths, 12,156 (15.0%) were home deaths. A home death was most frequent in ‘Circulatory diseases’ and ‘Cancer’, and associated with male sex, younger age, receiving domiciliary care and living alone. Only 2.3% of home deaths were from ‘Dementia’. In total, 41.9% of home deaths and 6.3% of all deaths were potentially planned home deaths. Potentially planned home deaths were associated with higher age, but declined in ages above 80 years for people who had municipal care. Living together with someone was associated with more potentially planned home deaths for people with municipal care.

**Conclusion:**

There are few home deaths in Norway. Our estimations indicate that even fewer people than anticipated have a potentially planned home death.

## Introduction

Most people wish to spend their last days of life in their own home and die at home [1]. Despite this, the proportion of home deaths in Western countries continues to decline [2]. Exceptions include Canada, the UK and the US, where home deaths have increased the last 15 to 25 years due to implementation of end-of-life programs and policy changes [2–4]. Home deaths increased from 19.3% in 1994 to 29.5% in 2004 in Canada; from 18.3% in 2004 to 20.8% in 2010 in the UK; and from 30.7% in 2000 to 33.5% in 2009 in Medicare beneficiaries aged 66 years and older in the US [2–4].

In Norway, home deaths have been declining, with only 14.3% of deaths taking place at home in 2015 [5, 6]. This is low compared with many other Western countries [7–16]. While home death is not desirable or possible for everyone, we need to know more about who dies at home and influencing factors in order to meet people’s preference of dying at home, as well as inform and improve policies. While we know the total number of home deaths, this does not describe the proportion of people who wished to die at home or the number of deaths that were planned or facilitated to take place at home. We cannot use registry data to estimate people’s preferences, but we can differentiate sudden, unplanned home deaths from home deaths where health and care service utilisation implies that resources were allocated to facilitate a home death.

In this study, our aim was first to describe factors associated with home deaths in Norway, and compare them with deaths in other locations. Secondly, we aimed to estimate how many home deaths may have been planned. Lastly, we wanted to analyse differences in individual characteristics between people where home deaths may have been planned, and people where home death did not appear to have been planned.

## Method

### Data source

We linked data from the Norwegian Cause of Death Registry (NCoDR) and National registry for statistics on municipal health and care services (IPLOS) covering all 83,434 deaths in Norway in 2012 and 2013. Individuals with missing information about place of death or sex (*n* = 2526) were excluded. The final study sample comprised 80,908 individuals.

Information on causes and place of death are registered in NCoDR [17]. In Norway, the doctor who examines the dead body completes the death certificate. This could be the treating general practitioner or institutional doctor, but also a doctor on night duty. The document is sent to the local county court/police, then to the Chief Municipal Medical Officer, before reaching NCoDR. The registry encompasses all residents, irrespective of whether they die in Norway or abroad, and since 2012 also information on deaths for non-residents. NCoDR has a high degree of coverage and completeness, with medical information on more than 98% of all deaths. Three quality assessments have ranked NCoDR in the second best group with “medium” and “medium-high” quality respectively, and lastly in the best group regarding quality. In all these three studies, the extensive use of unspecific codes served to lower the score. Few validation studies have been conducted [17].

IPLOS is a national registry for statistics on municipal health and care services. Since 2007 it has been the main data source for Norwegian municipal health and care statistics. It is compulsory for municipalities to register information on all persons who apply for or receive municipal health and care services, describing the person’s resources, need of assistance and services provided. Hospital admissions are also registered, but not used here due to poor data quality. Updates are continuously registered, and sent to the register annually. Data quality is assessed by comparison with information in other official statistics (KOSTRA – Municipality-State-Reporting) and reports returned to the municipalities [18, 19].

### Outcome measures

NCoDR provided information regarding cause of death, place of death, age, sex and place of residence (municipality population and centrality). IPLOS provided information regarding municipal services and household. We divided place of death into four categories: home; nursing home; hospital; other (abroad, under transportation to hospital, other). Cause of death was given by the European Shortlist for Causes of Death (EU Shortlist) [20]. Persons missing cause of death, with diagnoses removed due to privacy or with cause of death main diagnosis groups with a frequency of less than 5% of all deaths were labelled “other”. We grouped cause of death into eight categories: ‘Infectious/parasitic’; ‘Symptoms/signs/ill-defined’; ‘External’; ‘Cancer’ (including uncertain malignancy potential); ‘Dementia’; ‘Circulatory’; ‘Respiratory’; ‘Other’. Age at time of death was divided into seven groups (0–39, 40–49, 50–59, 60–69, 70–79, 80–89, 90+ years). Municipality centrality was defined as a municipality’s geographic location in relation to a centre with important central functions, where 0 is least central (rural) and 3 is most central (urban) [21]. Domiciliary care was coded ‘yes’ if an individual received practical help or home nursing at any time 0–90 days before death. Nursing home was coded ‘yes’ if an individual had a stay of any duration in a nursing home or rehabilitation facility 0–90 days before death. Individuals residing in assisted living accommodations with separate apartments were coded as living alone by IPLOS, while individuals in long-term institutional care were coded as cohabiting [18].

### Planned and unplanned home deaths

**Fig. 1:**
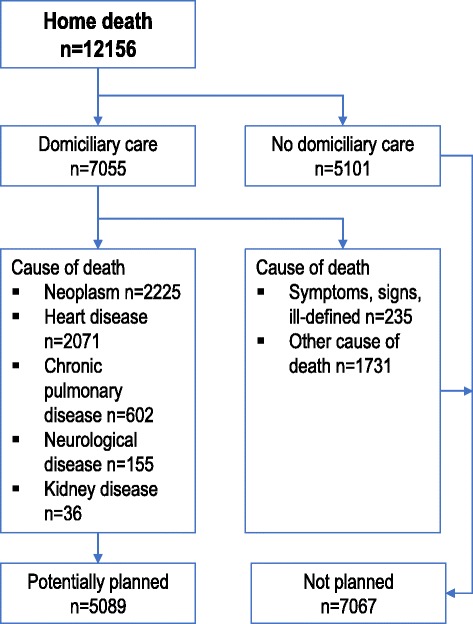
Algorithm for potentially planned and unplanned home deaths

We created an algorithm to estimate potentially planned and unplanned home deaths (Fig. 1). A home death was defined as unplanned if a person did not receive domiciliary care during the last ninety days before death or if a person who died at home with domiciliary care had cause of death ‘Symptoms/signs/ill-defined’. ‘Symptoms/signs/ill-defined’ were labelled unplanned, since people who have a planned home death would most likely have a known diagnosis of life-threatening disease later appearing as underlying cause of death in the death certificate; as opposed to people dying at home suddenly, but not unexpectedly of an unspecific cause. We defined a home death as potentially planned if a person had domiciliary care and a cause of death among the most likely diagnoses to receive palliative care. These were according to the EU Shortlist: ‘Cancer’ (2.), ‘Heart disease’ (7.1.2/7.1, 7.2.2, 7.4), ‘Chronic pulmonary disease’ (8.3.2), ‘Kidney disease’ (12.1) and ‘Neurological disease’ (6.1, 6.3) [20, 22]. Because the majority of people with dementia die in nursing homes, cause of death from ‘Dementia’ was not included [23]. The remaining home deaths were categorised as unplanned. The age group 0–39 years was given a lower detail level for cause of death than older age groups due to privacy. Cause of death was divided into six categories according to diagnoses most likely to receive palliative care: ‘Cancer’; ‘Circulatory’; ‘Respiratory’; ‘Kidney’; ‘Neurological’; ‘Other’.

### Analyses

We used frequency tables and Pearson Chi-square for comparisons. Multinomial logistic regression was used to compare death at home, in nursing home and hospital. Independent variables were sex, age, cause of death, municipality centrality, municipality population and domiciliary care. We did a similar analysis including type of household as explanatory variable in a subpopulation of people who received municipal care at any time (registered in IPLOS). Results are presented as adjusted relative risk ratios (RRRs) with 95% confidence intervals (CI) and *p*-values. xWe used logistic regression to compare potentially planned and unplanned home deaths. Independent variables were sex, age, municipality population, municipality centrality and nursing home stay (model 1). We did a separate analysis for people who received municipal care at any time (registered in IPLOS) with household as an explanatory variable (model 2). Unadjusted and adjusted odds ratios (ORs) with 95% CI and p-values are presented. Significance was accepted at the 5% level (*p* < 0.05) for all analyses. Analyses were conducted with STATA 14 (Stata Corp, College Station, TX).

## Results

### Describing the population

Among 80,908 deaths 12,156 (15.0%) were classified as home deaths. In total, more women died, with the majority (56.5%) dying in nursing homes (Table 1). While 18.1% of all men died at home, only 12.2% of all women died at home.

**Table 1:** Distribution of sociodemographic factors and municipal healthcare services by place of death in Norway 2012–2013.*

	Place of death									
	Home		Nursing home		Hospital		Other^a^		Total	
	n	%	n	%	n	%	n	%	n	%
All	12,156	100	39,345	100	26,962	100	2445	100	80,908	100
Sex										
Female	5175	42.6	23,904	60.8	12,613	46.8	598	24.5	42,290	52.3
Male	6981	57.4	15,441	39.2	14,349	53.2	1847	75.5	38,618	47.7
Age (years)										
0–39	549	4.5	70	0.2	796	3.0	426	17.4	1841	2.3
40–49	550	4.5	191	0.5	724	2.7	263	10.8	1728	2.1
50–59	1249	10.3	776	2.0	1877	7.0	389	15.9	4291	5.3
60–69	2381	19.6	2477	6.3	4553	16.9	564	23.1	9975	12.3
70–79	2538	20.9	5917	15.0	6212	23.0	386	15.8	15,053	18.6
80–89	3293	27.1	16,595	42.2	9134	33.9	338	13.8	29,360	36.3
90+	1596	13.1	13,319	33.9	3666	13.6	79	3.2	18,660	23.1
Cause of death										
Infectious/parasitic	1077	8.9	3562	9.1	2131	7.9	264	10.8	7034	8.7
Cancer	2648	21.8	10,728	27.3	8431	31.3	106	4.3	21,913	27.1
Dementia	274	2.3	4951	12.6	211	0.8	11	0.5	5447	6.7
Circulatory	4244	34.9	11,341	28.8	8692	32.2	727	29.7	25,004	30.9
Respiratory	952	7.8	4078	10.4	3117	11.6	65	2.7	8212	10.2
Symptoms/signs/ill-defined	603	5.0	343	0.9	39	0.1	340	13.9	1325	1.6
External causes	754	6.2	416	1.1	788	2.9	657	26.9	2615	3.2
Other	1604	13.2	3926	10.0	3553	13.2	275	11.2	9358	11.6
Household										
Cohabiting	3231	26.6	21,727	55.2	8692	32.2	240	9.8	33,890	41.9
Living alone	4038	33.2	15,341	39.0	8825	32.7	336	13.7	28,540	35.3
Missing^b^	4887	40.2	2277	5.8	9445	35.0	1869	76.4	18,478	22.8
Municipality population										
0–2000	425	3.5	1472	3.7	767	2.8	123	5.0	2787	3.4
2001–5000	1460	12.0	4733	12.0	2508	9.3	271	11.1	8972	11.1
5001–10,000	1795	14.8	5579	14.2	3381	12.5	354	14.5	11,109	13.7
10,001–50,000	4840	39.8	14,844	37.7	10,504	39.0	903	36.9	31,091	38.4
50,001-	3603	29.6	12,632	32.1	9683	35.9	746	30.5	26,664	33.0
Missing^b^	33	0.3	85	0.2	119	0.4	48	2.0	285	0.4
Municipality centrality^c^										
Least central	1531	12.6	5129	13.0	2850	10.6	346	14.2	9856	12.2
Less central	858	7.1	2664	6.8	1859	6.9	201	8.2	5582	6.9
Somewhat central	2431	20.0	7651	19.4	4954	18.4	393	16.1	15,429	19.1
Central	7303	60.1	23,816	60.5	17,180	63.7	1457	59.6	49,756	61.5
Missing^b^	33	0.3	85	0.2	119	0.4	48	2.0	285	0.4
Nursing home^d^										
Yes	1448	11.9	39,945	100.0	7945	29.5	139	5.7	48,877	60.4
No	10,708	88.1	0	0.0	19,017	70.5	2306	94.3	32,031	39.6
Domiciliary care^d^										
Yes	7055	58.0	16,455	41.8	15,456	57.3	479	19.6	39,445	48.8
No	5101	42.0	22,890	58.2	11,506	42.7	1966	80.4	41,463	51.2

*Pearson chi-square test: *p* < 0.001 for all categories

^a^Other place of death includes abroad, under transportation to hospital, other specified

^b^Not included in statistical analysis

^c^Classification based on geographical distance to centre with higher functions

^d^Service any time in the period 0–90 days before death

**Fig. 2:**
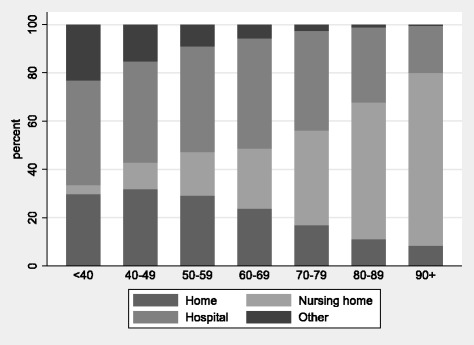
Distribution of place of death by age groups

Absolute number of home deaths was higher in the older age groups. However, within each age group, home death was more frequent for younger persons (Fig. 2). In people 0–69 years, 26.5% died at home, 19.7% in a nursing home, 44.6% in a hospital and 9.2% in other locations. Only 11.8% of people 70 years or older died at home, while 56.8% died in a nursing home, 30.1% in a hospital and 1.3% in other locations. The most common causes of death were ‘Circulatory diseases’, ‘Cancer’, ‘Respiratory diseases’, ‘Infectious diseases’ and ‘Dementia’ (Table 1). ‘Circulatory diseases’ (34.9%) and ‘Cancer’ (21.8%) were also the most frequent causes of death within the home death group, while only 2.3% were from ‘Dementia’.

A large proportion of deaths had missing information regarding household, largely reflecting people who never received municipal care (Table 1). For those who had services at any time, people living alone (14.2%) died at home more often than those living with others (9.5%). In total, 11.6% had a nursing home stay and 58.0% received domiciliary care in the last 90 days before dying at home. Of all persons who had a nursing home stay in the last 90 days before death, 3.0% died at home, while 17.9% of those receiving domiciliary care died at home.

### Comparing home deaths with deaths in nursing homes and hospitals

Multinomial regression showed that women had a lower relative risk than men for dying at home compared with nursing home (Table 2). We found an age gradient with more home deaths in younger age groups, except for people aged 90 years or older who had a higher relative risk for dying at home than in a hospital, but highest relative risk of dying in a nursing home. People who died from ‘Cancer’ and ‘Respiratory disease’ had lower relative risk of home death than ‘Circulatory disease’ compared with nursing home and hospital. People with ‘Dementia’ had higher relative risk of dying in nursing homes than home, but lower relative risk of dying in hospitals. Recipients of domiciliary care had higher relative risk of dying at home than in nursing homes or hospitals. A similar regression analysis with only persons who at any time had received municipal care (registered in IPLOS), including household as independent variable, did not alter main findings, but showed that people living with others had lower relative risk of home death compared with death in nursing home (RRR 0.77, 95% CI 0.73, 0.82) and hospital (RRR 0.90, 95% CI 0.87, 0.94).

**Table 2:** Adjusted relative risk ratio (RRR) for death at home versus nursing home, and home versus hospital^a^

	Home versus nursing home^a^			Home versus hospital^a^		
	Adjusted RRR	CI	p	Adjusted RRR	CI	p
Sex						
Female	0.67	0.64, 0.71	< 0.001	0.97	0.93, 1.02	0.223
Male	Ref.			Ref.		
Age						
0–39	49.76	38.34, 64.58	< 0.001	2.21	1.94, 2.51	< 0.001
40–49	19.77	16.55, 23.61	< 0.001	2.61	2.31, 2.96	< 0.001
50–59	11.71	10.55, 12.99	< 0.001	2.38	2.18, 2.60	< 0.001
60–69	7.20	6.68, 7.75	< 0.001	1.91	1.78, 2.04	< 0.001
70–79	2.71	2.55, 2.89	< 0.001	1.32	1.24, 1.41	< 0.001
80–89	Ref.			Ref.		
90+	0.55	0.52, 0.59	< 0.001	1.11	1.03, 1.19	0.006
Cause of death						
Infectious and parasitic diseases	0.95	0.88, 1.04	0.267	1.03	0.95, 1.12	0.461
Cancer	0.22	0.21, 0.24	< 0.001	0.48	0.45, 0.51	< 0.001
Dementia	0.23	0.20, 0.26	< 0.001	3.18	2.64, 3.82	< 0.001
Circulatory	Ref.			Ref.		
Respiratory	0.51	0.47, 0.56	< 0.001	0.60	0.55, 0.65	< 0.001
Symptoms/signs/ill-defined	3.84	3.29, 4.48	< 0.001	32.21	23.22, 44.71	< 0.001
External causes	1.92	1.66, 2.21	< 0.001	1.55	1.38, 1.74	< 0.001
Other	0.58	0.54, 0.63	< 0.001	0.76	0.71, 0.82	< 0.001
Municipality population						
0–2000	1.11	0.97, 1.28	0.135	1.67	1.45, 1.93	< 0.001
2001–5000	1.13	1.03, 1.24	0008	1.73	1.58, 1.93	< 0.001
5001–10,000	1.11	1.02, 1.20	0.014	1.54	1.42, 1.66	< 0.001
10,001–50,000	1.06	1.00, 1.13	0.033	1.30	1.23, 1.37	< 0.001
50,001-	Ref.			Ref.		
Municipality centrality						
Least central	0.95	0.87, 1.03	0.227	0.94	0.86, 1.03	0.198
Less central	1.07	0.97, 1 17	0.190	0.90	0.82, 0.99	0.024
Somewhat central	1.02	0.96, 1.09	0.465	1.01	0.95, 1.07	0.839
Central	Ref.			Ref.		
Domiciliary care						
Yes	2.70	2.58, 2.83	< 0.001	1.35	1.29, 1.42	< 0.001
No	Ref.			Ref.		

^a^Multinomial logistic regression with place of death as dependent variable. Number of observations 78,226

### Comparing potentially planned and unplanned home deaths

According to our algorithm for estimating potentially planned and unplanned home deaths (Fig. 1), 56.1% of home deaths and 8.4% of all deaths were potentially planned home deaths when we included everyone with domiciliary care but not ‘Symptoms/signs/ill-defined’ causes of death. In the full algorithm, including cause of death, 41.9% of home deaths and 6.3% of all deaths were potentially planned home deaths. Men had a higher proportion of both potentially planned and unplanned home deaths than women (Table 3). In people aged 50 years or older 44.5% of home deaths were potentially planned, but only 15.7% in those younger than 50 years. Living alone was more frequent in both potentially planned and unplanned home deaths, but more than half of unplanned home deaths had missing information regarding household, making comparisons uncertain. While 84.0% of ‘Cancer’ home deaths were potentially planned, this constituted only 10.2% of all ‘Cancer’ deaths. The proportion of potentially planned home deaths among all home deaths and total number of deaths for each diagnosis were 76.4% and 8.5% in ‘Neurological disease’, 69.2% and 7.4% in ‘Renal disease’, 63.2% and 7.3% in ‘Respiratory disease’ and 48.8% and 8.3% in ‘Circulatory disease’, respectively. Moreover, 72.1% of all home deaths and 12.9% of all deaths were potentially planned in recipients of domiciliary care.

**Table 3:** Comparing potentially planned home deaths with unplanned home deaths and all deaths^a^

	Potentially planned home death		Unplanned home death			All deaths	
	n	%	n	%	*p*	n	%
All	5089	100	7067	100		80,908	100
Sex					< 0.001		
Female	2458	48.3	2717	38.5		42,290	52.3
Male	2631	51.7	4350	61.5		38,618	47.7
Age (years)					< 0.001		
0–39	60	1.2	489	6.9		1841	2.3
40–49	113	2.2	437	6.2		1728	2.1
50–59	367	7.2	882	12.5		4291	5.3
60–69	887	17.4	1494	21.1		9975	12.3
70–79	1115	21.9	1423	20.1		15,053	18.6
80–89	1673	32.9	1620	22.9		29,360	36.3
90+	874	17.2	722	10.2		18,660	23.1
Cause of death^b^					< 0.001		
Cancer	2225	43.7	423	6.0		21,913	27.1
Circulatory	2071	40.7	2173	30.7		25,004	30.9
Respiratory	602	11.8	350	5.0		8212	10.2
Neurological	155	3.0	48	0.7		1819	2.2
Renal	36	0.7	16	0.2		489	0.6
Other	0	0.0	4057	57.4		23,471	29.0
Household					< 0.001		
Cohabiting	2273	44.7	958	13.6		33,890	41.9
Living alone	2366	46.5	1672	23.7		28,540	35.3
Missing^c^	450	8.8	4437	62.8		18,478	22.8
Municipality population					< 0.001		
0–2000	175	3.4	250	3.5		2787	3.4
2001–5000	651	12.8	809	11.4		8972	11.1
5001–10,000	826	16.2	969	13.7		11,109	13.7
10,001–50,000	2092	41.1	2748	38.9		31,091	38.4
50,001-	1337	26.3	2266	32.1		26,664	33.0
Missing^c^	8	0.2	25	0.4		285	0.4
Municipality centrality					< 0.001		
Least central	647	12.7	884	12.5		9856	12.2
Less central	377	7.4	481	6.8		5582	6.9
Somewhat central	1071	21.0	1360	19.2		15,429	19.1
Central	2986	58.7	4317	61.1		49,756	61.5
Missing^c^	8	0.2	25	0.4		285	0.4
Nursing home^d^					< 0.001		
Yes	860	16.9	549	7.8		46,638	57.6
No	4229	83.1	6518	92.2		34,270	42.2
Domiciliary care^d^					< 0.001		
Yes	5089	100.0	1966	27.8		39,445	48.8
No	0	0.0	5101	72.2		41,463	51.2

^a^*P* Pearson chi-square test for planned and unplanned home deaths

^b^Cause of death was divided into five categories according to diagnoses most likely to receive palliative care, the rest were labelled other

^c^Not included in statistical analysis

^d^Service any time in the period 0–90 days before death

Table 4 compares two regression models of potentially planned versus unplanned home deaths. In model 1, including all home deaths, women had higher odds than men for having a potentially planned home death, but in model 2, which was restricted to include people who at any time had received municipal care, women had lower odds for having a potentially planned home death. Higher age was associated with more potentially planned home deaths, but in model 2 ages 60–79 years had the highest odds, with declining odds in older age groups. Municipalities with less than 50,000 inhabitants were consistently associated with more potentially planned home deaths, of which municipalities with 5001–10,000 inhabitants had the highest odds. A nursing home stay during the last 90 days before death was associated with higher odds for a potentially planned home death in model 1, but had lower odds in model 2. Living together with someone increased the odds for a potentially planned home death in model 2.

**Table 4:** Odds ratio (OR) for potentially planned home death compared with unplanned home death ^a^

	Unadjusted			Model 1			Model 2		
	OR	CI	*p*	Adjusted OR	CI	*p*	Adjusted OR	CI	*p*
Sex									
Female	1.50	(1.39, 1.61)	< 0.001	1.29	(1.20, 1.40)	< 0.001	0.90	(0.81, 0.99)	0.039
Male	Ref.			Ref.			Ref.		
Age									
0–39	0.12	(0.09, 0.16)	< 0.001	0.14	(0.11, 0.18)	< 0.001	0.19	(0.13, 0.26)	< 0.001
40–49	0.25	(0.20, 0.31)	< 0.001	0.29	(0.23, 0.37)	< 0.001	0.41	(0.31, 0.54)	< 0.001
50–59	0.40	(0.35, 0.46)	< 0.001	0.46	(0.40, 0.53)	< 0.001	0.71	(0.59, 0.87)	0.001
60–69	0.57	(0.52, 0.64)	< 0.001	0.65	(0.58, 0.72)	< 0.001	1.26	(1.07, 1.48)	0.005
70–79	0.76	(0.68, 0.84)	< 0.001	0.82	(0.74, 0.91)	< 0.001	1.32	(1.14, 1.52)	< 0.001
80–89	Ref.			Ref.			Ref.		
90+	1.17	(1.04, 1.32)	0.009	1.10	(0.97, 1.25)	0.122	0.80	(0.70, 0.92)	0.002
Municipality population									
0–2000	1.19	(0.97, 1.46)	0.102	1.29	(1.02, 1.64)	0.032	1.40	(1.01, 1.95)	0.043
2001–5000	1.36	(1.21, 1.54)	< 0.001	1.36	(1.17, 1.58)	< 0.001	1.40	(1.15, 1.71)	0.001
5001–10,000	1.44	(1.29, 1.62)	< 0.001	1.46	(1.28, 1.67)	< 0.001	1.47	(1.22, 1.75)	< 0.001
10,001–50,000	1.29	(1.18, 1.41)	< 0.001	1.27	(1.15, 1.40)	< 0.001	1.27	(1.12, 1.44)	< 0.001
50,001-	Ref.			Ref.			Ref.		
Municipality centrality									
Least central	1.06	(0.95, 1.18)	0.321	0.85	(0.74, 0.99)	0.031	0.94	(0.78, 1.15)	0.570
Less central	1.13	(0.98, 1.31)	0.086	0.99	(0.85, 1.16)	0.923	1.02	(0.82, 1.26)	0.868
Somewhat central	1.14	(1.04, 1.25)	0.006	0.99	(0.89, 1.10)	0.877	0.99	(0.86, 1.14)	0.904
Central	Ref.			Ref.			Ref.		
Nursing home									
Yes	2.44	(2.18, 2.73)	< 0.001	2.00	(1.78, 2.25)	< 0.001	0.78	(0.68, 0.88)	< 0.001
No	Ref.			Ref.			Ref.		
Household									
Cohabiting	1.68	(1.52, 1.85)	< 0.001				1.62	(1.46, 1.80)	< 0.001
Living alone	Ref.						Ref.		

^a^unadjusted, adjusted model 1 for all home deaths (*n* = 12,123) and adjusted model 2 for persons who had received municipal care (*n* = 7261)

## Discussion

### Main findings

This population-based registry study showed that home death in Norway was most frequent in ‘Circulatory disease’ and ‘Cancer’, and associated with male sex, younger age, receiving domiciliary care and living alone. In total, 41.9% of home deaths and 6.3% of all deaths were potentially planned. Potentially planned home deaths were associated with higher age, but declined in ages above 80 years for people who had municipal care. Living together with someone was associated with more potentially planned home deaths for people with municipal care.

### Strengths and limitations

To our knowledge, this is the first study to estimate number of potentially planned home deaths by using population-based registry data. The use of routinely collected data, minimises the burden on patients and caregivers, associated with primary data collection in end-of-life care context [24]. The use of death certificates is similar across countries and comparable. NCoDR provides cause of death for more than 98% of all deaths in Norway, but has high use of unspecific cause of death codes increasing the risk of misclassification. Additionally, few diagnoses are verified by autopsy [17]. The study accounts for domiciliary care and nursing home admissions prior to death, but it is a limitation that we do not have information regarding hospital admissions. A methodological limitation is that we have no exact data on planned home deaths, but use an indirect approach by an algorithm. However, there is no registry based information source available as an alternative. A limitation for some analyses is that information regarding whether a person lived alone or together with others was only available for people who had received municipal care. Our definition of a potentially planned home death may also have led to deaths from acute illness being classified as potentially planned. Moreover, we cannot exclude that persons with other diagnoses than included in our definition may have had planned home deaths.

### Few potentially planned home deaths

Our data cannot give information regarding a person’s preference for dying at home. However, we will argue that our estimation of potentially planned home deaths in Norway is a valid indication. Planned home death is not feasible without the support of domiciliary care and not probable when cause of death is unknown. Even if there is no preference of dying at home, domiciliary care in itself signals facilitation of more time at home and increases the probability of dying at home [25]. Thus, the highest proportion of home deaths that was not random, and could potentially have been planned, was 56.1% or 8.4% of all deaths in our model. However, it is likely that the proportion of potentially planned home deaths is even lower in Norway than our final estimation (41.9% of home deaths and 6.3% of all deaths), as palliative care is mostly given to people with cancer [22, 26].

Most research regarding planned home deaths are interventions trying to enable more home deaths, with little data on the actual rate of planned home deaths before the intervention [27]. We found that only 49% of home deaths from ‘Circulatory disease’ were potentially planned, while 84% of home deaths from ‘Cancer’ were potentially planned. This indicates a large proportion of sudden or unexpected home deaths from ‘Circulatory disease’, but could also indicate inequality between these groups in recognition of palliative care needs [28, 29].

### Comparison with previous research

Previous studies have shown that the proportion of home deaths is associated with low functional status, preferences on place of death, home care and its intensity, living with relatives, extended family support, home palliative care, not living in urban areas, higher socio-economic status and being male [8, 14, 16, 25]. Other factors influencing home death are culture, ethnicity and number of hospital and nursing home beds. The relationship with age is more complicated [8, 14, 16, 25].

We found that living alone was associated with more home death. This should be interpreted with caution as we had a large proportion of missing data, but could indicate a large proportion of unexpected home deaths for people living alone. However, in a subpopulation of people who had received municipal care, potentially planned home deaths were associated with living together with someone.

There is no consensus definition on rurality, but there is consistency in associations with place of death across definitions [30]. We did not find any association with our definition of rural areas [21], but municipalities with fewer inhabitants were associated with more home death compared to hospital and also with more potentially planned home deaths. This indicates that other factors than travelling distance to hospitals influence proportion of home deaths. Home nursing coverage could be a contributing factor, as smaller municipalities in Norway have better home nursing coverage than larger municipalities. The largest cities have higher coverage of long-term care in institutions and the lowest coverage of home nursing, which could shift end-of-life care to nursing homes instead of home [31].

Our results also showed that fewer women died at home than men. The association was significant when compared to nursing home, similar to a Swedish study [9]. A possible explanation could be that women care for their spouses and live longer, but the association was present also after adjusting for age and living together with someone. Another explanation could be that men have more sudden and unexpected home deaths, as only men had home deaths from external causes of death or symptoms/signs/ill-defined and more unplanned home deaths from circulatory disease. Still, men had higher odds of a potentially planned home death in the subpopulation who had received municipal care.

Like many other countries, Norway has experienced declining home death rates and a shift from hospital to nursing home deaths [6, 8, 14] . This is partly due to population aging but also end-of-life care policy [2–4, 6]. Incongruence between preferred and actual place of death is common, especially for people with non-malignant disease [29]. Dying in their preferred place is considered a quality indicator of care, and should together with evidence that a majority of people prefer to die at home, be reflected in future planning of palliative care services [1, 32].

Transitions in the last phase of life is another important factor to consider when evaluating quality of end-of-life care. Transitions in the last months and days before death are common, with more than half of dying people having at least one transition [33, 34]. Transitions are shown to be more common in home-dwelling people, where about half have a final transition from home to hospital [33, 34]. Groff et al. found an inverse relationship between number of days in domiciliary care and days spent at home in the last six months before death, interpreted as doing more of one thing led to doing more in other areas as well, and did not necessarily improve patient-centred goals [35]. Although home death will never be a goal or possible for all dying people, a more person-centred goal like “days spent at home” could change the perspective of both the dying person and caregivers and lead to increased time spent at home in the final phase of life, and together with palliative homecare reduce symptom burden and increase chances of home death according to the person’s own wishes [27, 36, 37].

## Conclusions

This registry based study from Norway shows that home death is relatively infrequent, and by an indirect algorithm-based definition, we have shown that less than half of them are potentially planned home deaths. Future research should investigate how many deaths that are actually planned to take place at home, and the achievement of this goal. There is also a need to understand the transitions in the last phase of life and whether the place of death corresponds to the patients’ wish, and how palliative homecare influences such outcomes.

## Abbreviations

EU Shortlist: European Shortlist for Causes of Death; RRR: Relative risk ratio
IPLOS: National registry for statistics on municipal health and care services
NCoDR: Norwegian Cause of Death Registry
